# Febrile patients admitted to remote hospitals in Northeastern Kenya: seroprevalence, risk factors and a clinical prediction tool for Q-Fever

**DOI:** 10.1186/s12879-016-1569-0

**Published:** 2016-06-03

**Authors:** J. Njeru, K. Henning, M. W. Pletz, R. Heller, C. Forstner, S. Kariuki, E. M. Fèvre, H. Neubauer

**Affiliations:** Institute of Bacterial Infections and Zoonoses, Friedrich-Loeffler-Institut, 07743 Jena, Germany; Center for Infectious Diseases and Infection Control, Jena University Hospital, 07740 Jena, Germany; Centre for Microbiology Research (CMR), Kenya Medical Research Institute, P. O. Box 19464-00202, Nairobi, Kenya; Institute for Molecular Cell Biology, Center for Molecular Biomedicine, Friedrich Schiller University of Jena, 07745 Jena, Germany; Department of Medicine I, Division of Infectious Diseases and Tropical Medicine, Medical University of Vienna, 1090 Vienna, Austria; Institute of Infection and Global Health, University of Liverpool, Liverpool, UK; International Livestock Research Institute (ILRI), Road, P.O. Box 30709-00100, Nairobi, Kenya

**Keywords:** Seroprevalence, Epidemiology, *Coxiella burnetii*, Q fever, Kenya

## Abstract

**Background:**

Q fever in Kenya is poorly reported and its surveillance is highly neglected. Standard empiric treatment for febrile patients admitted to hospitals is antimalarials or penicillin-based antibiotics, which have no activity against *Coxiella burnetii*. This study aimed to assess the seroprevalence and the predisposing risk factors for Q fever infection in febrile patients from a pastoralist population, and derive a model for clinical prediction of febrile patients with acute Q fever.

**Methods:**

Epidemiological and clinical data were obtained from 1067 patients from Northeastern Kenya and their sera tested for IgG antibodies against *Coxiella burnetii* antigens by enzyme-linked-immunosorbent assay (ELISA), indirect immunofluorescence assay (IFA) and quantitative real-time PCR (qPCR). Logit models were built for risk factor analysis, and diagnostic prediction score generated and validated in two separate cohorts of patients.

**Results:**

Overall 204 (19.1 %, 95 % CI: 16.8–21.6) sera were positive for IgG antibodies against phase I and/or phase II antigens or *Coxiella burnetii* IS1111 by qPCR. Acute Q fever was established in 173 (16.2 %, 95 % CI: 14.1–18.7) patients. Q fever was not suspected by the treating clinicians in any of those patients, instead working diagnosis was fever of unknown origin or common tropical fevers. Exposure to cattle (adjusted odds ratio [aOR]: 2.09, 95 % CI: 1.73–5.98), goats (aOR: 3.74, 95 % CI: 2.52–9.40), and animal slaughter (aOR: 1.78, 95 % CI: 1.09–2.91) were significant risk factors. Consumption of unpasteurized cattle milk (aOR: 2.49, 95 % CI: 1.48–4.21) and locally fermented milk products (aOR: 1.66, 95 % CI: 1.19–4.37) were dietary factors associated with seropositivity. Based on regression coefficients, we calculated a diagnostic score with a sensitivity 93.1 % and specificity 76.1 % at cut off value of 2.90: fever >14 days (+3.6), abdominal pain (+0.8), respiratory tract infection (+1.0) and diarrhoea (−1.1).

**Conclusion:**

Q fever is common in febrile Kenyan patients but underappreciated as a cause of community-acquired febrile illness. The utility of Q fever score and screening patients for the risky social-economic and dietary practices can provide a valuable tool to clinicians in identifying patients to strongly consider for detailed Q fever investigation and follow up on admission, and making therapeutic decisions.

## Background

Q fever is an acute (on occasion chronic) zoonotic disease of global public health importance. The disease is caused by the obligate Gram-negative bacterium *Coxiella (C.) burnetii* [1]. Domestic animals such as cattle, sheep and goats are the main reservoirs of *C. burnetii* which can infect a large variety of animals, humans, and arthropods [2]. Infection in humans usually occurs by inhalation of contaminated aerosols, consumption of contaminated unpasteurized dairy products, direct contact with contaminated milk, urine, feces, or semen of infected animals, and tick bites [3]. Clinical presentation is nonspecific and highly variable ranging from asymptomatic infection (60 %) or self-limiting febrile illness associated with fatigue, headache, general malaise, myalgia, arthralgia, to atypical pneumonia (rapidly progressive courses may occur) and/or hepatitis. Less frequent manifestations include endocarditis, osteomyelitis and aseptic meningitis. About 1–2 % of acute symptomatic cases may develop chronic disease [4, 5]. Q fever is considered to be an occupational disease of people who have intimate contact with animals or their products such as veterinarians, farmers, abattoir workers, and laboratory workers [4, 6].

Acute Q fever in humans is confirmed when a patient present with clinically compatible symptoms and detection of the *C. burnetii* by at least one of the following diagnostic tests; cultivation, detection of *C. burnetii* DNA from any clinical specimens (usually blood or respiratory secretions), detection of *C. burnetii* in a clinical specimen by immunohistochemistry (IHC), seroconversion or a fourfold increase from non-negative titer sera [7]. In the absence of positive culture, IHC or PCR results, and when acute and convalescent serum samples cannot be obtained, elevated phase II IgG antibodies level by ELISA or positive indirect immunofluorescence assay (IFA) (IgG phase II ≥1:128) in a patient who has been ill longer than 1 week is laboratory supportive of acute Q fever infection while IgG phase I titer ≥1:800 is seen in chronic patients [2, 7–9].

Q fever is a notifiable disease in many developed countries, but it is poorly reported in sub-Saharan Africa and its surveillance is highly neglected [10]. Available reports from previous studies show remarkable high seroprevalence in the African countries with intensive livestock production systems [11–13]. Pastoralist communities are particularly at high risk of pathogen exposure because of their itinerant lifestyle and highly conserved traditions that make them more likely to consume unboiled milk products and raw meat from infected animals. They are also less likely to protect themselves when handling animal birth products and vaginal discharges after abortion or full-term parturition [14, 15]. Despite these, few studies have investigated in detail the risk factors or the reasons for variation of prevalence in the diverse agro-ecological African settings [10]. This lack of attention is mainly caused by lack of data and the perceived low clinical relevance of Q fever in relation to other endemic fevers [16, 17].

In Kenya, Q fever in humans was first reported in hospitalized patients in 1950s [18–20]. A serosurvey by Vanek and Thimm, (1976) detected seroprevalences ranging from 10 to 35.8 % in patients from five provinces of Kenya [21]. An outbreak of Q fever involving safari travelers in a game park was described in 2000 in which 4 (8 %) of fifty travelers contracted the disease [22]. A recent study in a rural hospital in western Kenya demonstrated IgG antibodies to *C. burnetii* antigens in 30.9 % of acute febrile illness (AFI) patients. In addition, acute Q fever was detected in 3 % of patients diagnosed with acute lower respiratory infections (ALRI) in the same hospital [17]. Among domestic animals in Kenya, prevalence of *Coxiella* antibodies was reported ranging from 7.4 to 51.1 % in cattle, 6.7 to 20 % in sheep, 20 to 40 % in goats, and 20 to 46 % in camels [17, 20, 21, 23].

There is emerging evidence of *C. burnetii* as a cause of non-malaria febrile illness and community acquired pneumonia in many African countries including Kenya [16, 17, 24–27]. Many etiologies of febrile illnesses are difficult to distinguish from one another clinically and reliable laboratory diagnostic facilities are often limited in developing countries, thus clinical management of such illness is often driven by syndrome-based local guidelines employing empiric treatment [28]. This makes timely and accurate diagnosis or management of the neglected diseases such Q fever an important challenge to clinicians [29]. A systematically collected data on Q fever burden estimates and risk factor analysis is needed to support development of targeted interventional control policies. In addition, a simple clinical algorithm with high diagnostic sensitivity and specificity remains an important tool for timely detection, and prevention of morbidity associated with Q fever in regions where laboratory facilities are lacking.

This study aimed to estimate the seroprevalence of Q fever in patients with febrile illness seeking treatment at two hospitals in the Northeastern province of Kenya and investigate the risk factors for seropositivity. We also evaluated the usefulness of clinical signs and symptoms in predicting Q fever outcome. We hypothesized that a clinical prediction score system with high diagnostic accuracy would be of benefit to clinicians who work in resource scarce settings in making decisions on febrile patients to strongly consider for Q fever investigation during initial diagnosis, and when making empirical therapeutic decisions. This hypothesis was tested using derivation and validation cohorts.

## Methods

### Study area

**Fig. 1:**
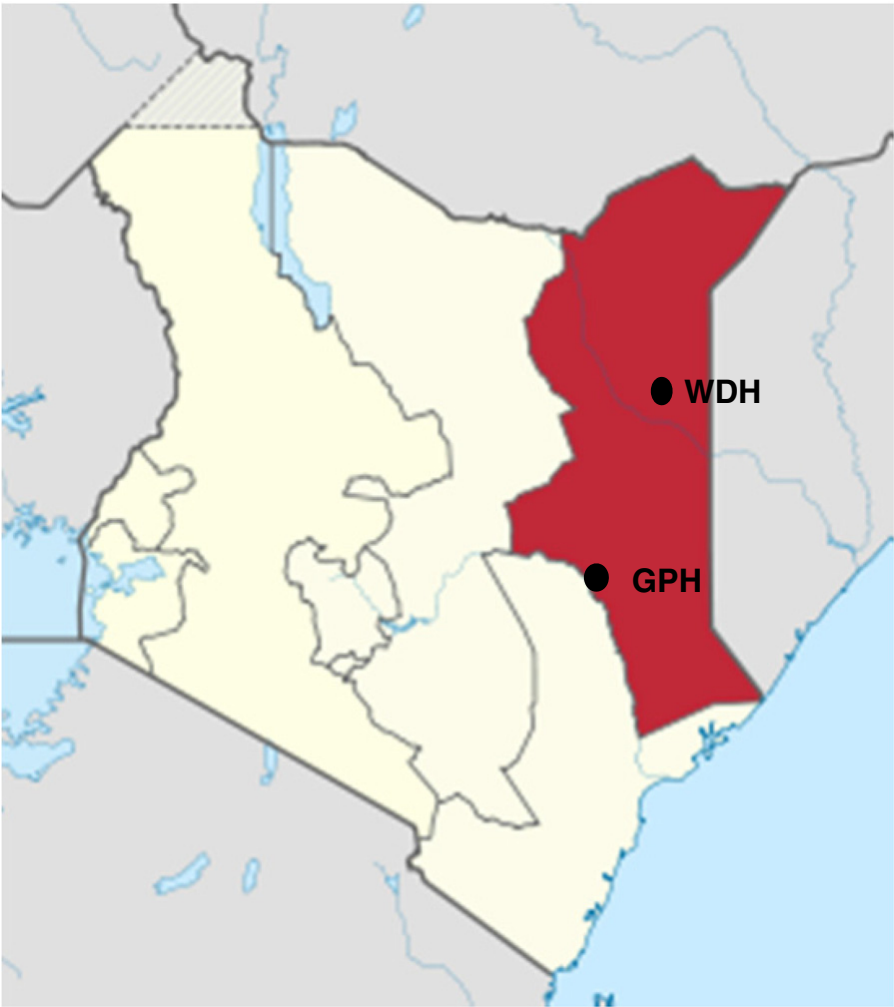
Map of Kenya showing Northeastern Province of Kenya (shaded area). The location of Garissa Provincial Hospital (GPH) and Wajir District Hospital (WDH) are marked with black dots

The study was conducted at Garissa Provincial Hospital (GPH) and Wajir District Hospital (WDH) of Northeastern province of Kenya (Fig. 1). GPH has 224 beds and serves an estimated population of 623,060 individuals, whereas WDH has 120 beds serving about 661,941individuals. These health facilities are also the main referral hospitals of the Garissa and Wajir counties respectively. The province has an arid and semi-arid lands (ASAL) climate and is almost exclusively inhabited by the Somali ethnic groups who are predominantly nomadic pastoralists. Livestock keeping is the main economic activity in the region. The common diet for the communities in the province is meat and milk [15, 30].

Drinking of raw milk is common in this population due to deep-rooted cultural traditions. It is believed that boiling milk reduces the nutritional value of milk. Milk is also mixed from several animals in common containers. From this milk, fermented products (local name ‘susac’) are prepared. Susac would then be sold at the nearest open market places and nomadic camp sites [15, 31]. This province was selected because public health support and disease surveillance has been extremely limited and outbreaks of re-emerging diseases have been reported in the province before [32–34].

### Study participants and procedures

The hospital-based study was conducted between June 2014 and January 2015. Acute febrile patients presenting to the outpatient departments of the two hospitals were systematically assessed for enrollment. The inclusion criteria were: a clinical history of acute febrile illness (AFI) characterized by fever (higher than or equal to 38 °C) and at least one of the following clinical symptoms: headache, chills, myalgia, arthralgia, general body malaise, and acute lower respiratory illness (ALRI). ALRI was defined as new onset of cough, difficulties when breathing or chest pain and fever [17]. A study clinician then collected demographic information, obtained clinical history and performed physical examination of the enrolled participants. The clinical diagnosis made by the attending hospital clinician was also recorded. Exclusion criteria were: patients younger than 18 years not accompanied by a guardian or parents, patients who had an already established diagnosis and those who were severely sick and were considered not able to provide informed consent. Patients who declined to participate were also excluded.

### Sample and data collection

Demographic information and data on putative social-economic and dietary characteristics of the patients were collected using pre-tested questionnaires. Risk factors were assessed in form of a mix of closed- and open-ended questions including the following data: (a) living in a livestock keeping household/nomad camp or involvement in animal husbandry activities for at least 2 days a week (hereafter referred to as ‘regular contact with animals’), (b) animal species involved, and (c) the type of contact with animals or their products i.e. help in animal parturition, contact with aborted materials, grazing animals, livestock trade, handling raw milk, and animal slaughter. Participants were also asked if they shared their house with animals and if they were involved in manure preparation. The food variables assessed were: (i) consumption of raw milk, (ii) locally fermented milk products (susac), (iii) drinking of animal blood and (iv) consumption of uncooked meat.

Following cleansing of the skin with isopropyl alcohol and povidone iodine, five milliliters of venous blood were collected aseptically into empty 10 ml serum BD vacutainer tubes® (Becton, Dickson and Company, USA). The blood samples were stored at room temperature for 45 min to allow blood clotting. The samples were then centrifuged at 2500 rpm for 5 min and serum was separated. Sera were aliquoted into cryo-vials before shipping to Friedrich-Loeffler-Institut (FLI) for Q fever laboratory analysis.

### Serological analysis

Sera were analyzed for IgG antibodies against phase I and phase II antigens using serion ELISA classic test kits (Virion\Serion, Würzburg, Germany) and evaluated according to the manufacturers’ recommendations. For phase I, samples with an optical density (OD) >10 % above OD of the cut-off sera were scored as positive, those with ODs >10 % below OD of the cut-off sere were considered as negative, in between samples were denoted as borderline. For phase II, the cut off value was calculated on the basis of the standard curve corrected by the mean of the extinction of the standard serum according to the manufacturers’ instructions. A result of <20 U/ml was regarded as negative, 20–30 U/ml borderline, and >30 U/ml as positive.

The seropositive samples and those with inconclusive results were confirmed with a commercial IFA for detection of IgG antibodies against *C. burnetii* phase I and phase II (Fuller Laboratories, USA). The assay titers were started at 1:16 and results interpreted according to the manufacturers’ instructions. Samples with *C. burnetii* phase II antibodies titers of >1:128 were scored as acute Q fever positive, while those with phase I antibodies titers of ≥1:800 were regarded as positive for chronic Q fever. The samples that were found to be equivocal in the repeat tests were re-tested at the Q fever consulting laboratory Baden-Wurttemberg, State Health Office, Germany.

### DNA extraction from samples

DNA isolation was done using the High Pure PCR Template preparation commercial Kit™ (Roche Diagnostics, Germany) according to the manufacturer’s instructions. Purity and concentration of DNA was tested using a Nano Drop ND-1000 UV-vis spectrophotometer (Nano Drop Technologies, Wilmington, USA), and DNA samples were stored at −20 °C until analyzed.

### Quantitative Real-time PCR assay (qPCR)

Detection of *C. burnetii* DNA was performed with a TaqMan based qPCR assay targeting the repetitive element IS1111 as described by Klee *et al*. [35] using a Stratagene Mx3000P v 4.01Thermocycler (Agilent Technologies, Santa Clara, USA). No template control (NTC) and tenfold serial dilution of cloned IS1111 gene plasmid fragments ranging from 1 × 10^0^ to 1 × 10^5^ plasmid copy numbers were used for *Coxiella* DNA quantification and sensitivity control of the assay. Cycling conditions were as follows: one pretreatment cycle at 50 °C for 2 min, initial denaturation at 95 °C for 10 min, followed by 50 cycles at 95 °C for denaturation for 15 s, and 30 s for annealing and elongation at 60 °C. No internal amplification control (IAC) was used in the procedure to ensure high sensitivity. Cycle threshold (Ct) values ≤ 36 cycles were interpreted as positive. The threshold was calculated automatically by the instruments’ software based on six 10 fold serial dilutions of *C. burnetii* DNA in negative human sera assessed simultaneously in a single run.

### Case definitions

Acute Q fever case was defined as a patient with compatible clinical evidence criteria and laboratory supportive results for acute Q fever illness, reflected by elevated IgG phase I and phase II antibodies by ELISA and confirmed by *C. burnetii* phase II antibodies titers of >1:128 by IFA assay or detection of *Coxiella* DNA by qPCR. Chronic case was defined as those with elevated IgG phase I antibodies by ELISA and phase I antibodies titers of ≥1:800 by IFA assay. The case definition criteria was based on a combination of recommendations of the kits manufacturer and as proposed by EFSA [2], CDC [7], and previous authors [8, 9, 36]. In all cases, *Salmonella* spp, *Brucella* spp, and *Plasmodium* spp were excluded. For statistical analysis, seropositivity was defined as any participant who met the criteria for acute or chronic Q fever.

### Data management and statistical analysis

Questionnaire results and serological data were entered into Microsoft excel 2010 spreadsheet and exported to SPSS Statistics software® (Armonk, IBM Corp, USA. v.20) and verified against the paper questionnaires and laboratory sheets for consistency and completeness. The Pearson’s *χ*^2^test or Fisher’s exact test was used to determine differences in Q fever seroprevalence among demographic groups. Statistical significance was set at *P* value < 0.05.

Multivariate logistic regression models were built to assess clinical features, and plausible socio-demographic, economic and dietary characteristics of the patients associated with Q fever seropositivity using stepwise backward analysis procedure. Briefly, univariate analysis was performed for all covariates in each model and pair-wise collinearity assessed using Spearman rank correlation. A pair of the variables was considered collinear if the correlation coefficient was equal or greater than 0.8. Multi collinearity was then assessed for all potential variables by estimating the variance inflation factor (cut-off 5) and tolerance (cut-off 0.2) levels using collinearity diagnostics function (IBM Corp, USA. v.20). The variables were then droped or aggregated as appropriate.

The candidate covariates with *p* < 0.2 using (Wald test) were fitted into the multivariable model and those exhibiting the highest p-values (Wald test) were removed one at a time from the model until all the retained variables had *p* < 0.05. The eliminated variables were re-screened for confounding and were retained if their inclusion caused 20 % or more change to the coefficients of one or more of the retained variables. Age was considered to be a biologically important variable and thus was kept in the final model. Finally, both the clinical and putative socio-economic and dietary characteristics from the two sub models were combined in a multivariate analysis to identify the independent risk determinants for Q fever seropositivity. The Hosmer-Lemeshow test was used for assessing final models’ goodness of fit (GOF). The area under the ROC curve was also generated to assess the predictive ability of the final best model.

To derive and evaluate a predictive clinical model best-fit for identification of a subgroup of patients with high likelihood of acute Q fever, the patients were randomly assigned to derivation set and validation set using random number generated using the SPSS random number generator function (IBM Corp, USA. v.20). The derivation set used to fit the model composed of 65 % of the sample (*n* = 707) while the validation set used to validate the model was made up of 35 % of the sample (*n* = 360) [37]. A clinical prediction score “Q fever score” was then generated using the β coefficients of the final best fit model and the discriminatory power of this score evaluated by the area under the receiver operating characteristics (ROC) curve at the 95 % confidence interval (CI). Then a cut-off value to estimate the diagnostic sensitivity and specificity in the validation set was selected [38].

## Results

### Study population

A total of 1067 participants (WDH: 536 and GPH: 531) were enrolled in the study. The study participants were fairly distributed between male and female (45.6 vs 54.4 %). Nine hundred thirty-six participants were adults (>18 years), of which 963 (90.3 %) were of Somali ethnicity. Whereas most of the patients did not have any formal education (*n* = 661; 61.9 %), nearly half (*n* = 448; 42.0 %) had knowledge about zoonosis but only 5 (0.5 %) reported prior knowledge of Q fever. Eight hundred eighty-five patients reported regular contact with at least one animal species. Out of these, the Somali ethnic group reported more contacts (*n* = 818; 84.9 %) than other ethnic groups (*n* = 67; 64.4 %). Demographic characteristics of patients as well as the number positive for acute Q fever are shown in Table 1.

**Table 1:** Demographic characteristics of study participants and numbers positive for acute Q fever among the febrile patients, Northeastern Kenya

Variable	Total Number (%)	IgG PII positive (*N* = 1067)	IgG PI positive (*N* = 1067)	IgG PI/PII positive (*N* = 1067)	qPCR positive (*N* = 448)	Q fever positive^a^ (%) (*N* = 1067)	95 % (CI)	P-value
Age Group (years)								
0–18	131 (12.3)	16	15	18	0	17 (13.0)	8.0–20.2	0.283
18+	936 (87.7)	165	160	176	10	156 (16.7)	14.4–19.3	
Total	1067 (100 %)	181 (17.0 %)	175 (16.4 %)	194 (18.2 %)	10 (2.2 %)	173 (16.2 %)	14.1–18.7	-
Gender								
Female	580 (54.4)	102	98	109	4	96 (16.6)	13.7–22.9	0.736
Male	487 (45.6)	79	77	85	6	77 (15.8)	12.6–19.4	
Occupation								
Herder	678 (63.5)	140	138	150	7	134 (19.8)	16.9–23.1	<0.001
Civil servant	142 (13.3)	10	8	10	0	9 (6.3)	3.1–12.2	
General merchandise	74 (6.9)	8	7	9	0	6 (8.1)	3.3–17.4	
Livestock trader	59 (5.5)	15	14	16	2	15 (25.4)	15.4–38.7	
Student	62 (5.8)	4	5	5	0	5 (8.1)	3.0–18.5	
Others	52 (4.9)	4	3	4	1	4 (7.7)	2.5–19.4	
Education Level								
None	661 (61.9)	129	132	141	7	130 (19.7)	16.8–22.9	<0.001
Post-secondary	114 (10.7)	12	10	12	0	10 (8.8)	4.5–15.9	
Primary	206 (19.3)	36	29	37	2	30 (14.6)	10.2–20.3	
Secondary	86 (8.1)	4	4	4	1	3 (3.5)	1.1–9.8	
Residence county								
Garissa	531 (49.7)	86	84	93	6	83 (15.6)	12.7–19.1	0.607
Wajir	536 (50.3)	95	91	101	4	90 (16.8)	13.8–20.3	
Ethnic groups								
Somali	963 (90.3)	173	167	188	8	165 (17.1)	14.8–19.7	0.004
Others	104 (9.7)	8	8	6	2	8 (7.7)	3.6–15.4	
Zoonosis knowledge								
Yes	448 (42.0)	62	61	67	2	58 (12.9)	10.0–16.5	
No	619 (58.0)	119	114	127	8	115 (18.6)	15.6–21.9	0.012
Q Fever knowledge								
Yes	5 (0.5)	0	0	0	0	0	-	-
No	1062 (99.5)	181	175	194	10	173 (16.2)	14.1–18.7	

*CI* confidence interval, *IgG PII/I* IgG antibodies against phase II/I *Coxiella* antigens

^a^Patients meeting case definition for acute Q fever by serology or *Coxiella* DNA detection by qPCR

Majority (*n* = 689; 64.6 %) of the participants reported regular contact with goats whilst less than half had contact with cattle (*n* = 465; 43.6 %) and sheep (*n* = 392; 36.7 %). Only 112 (10.5 %) reported regular exposure to camels. The type of exposure included; animal grazing (*n* = 717; 67.2 %), handling of raw milk (*n* = 597; 56 %), and animal slaughter (*n* = 382; 35.8 %). One hundred fifty-eight (14.8 %) patients reported to help in animal parturition and 44 (4.1 %) reported exposure to aborted or after birth animal materials. Consumption of raw milk was common among the participants. Almost all participants of Somali origin (*n* = 907; 94.2 %) reported consumption of raw milk. Less than a half (*n* = 38; 36.5 %) of other ethnic groups reported to do so. Consumption of raw camel milk (*n* = 943; 88.4 %), raw cattle milk (*n* = 331; 31 %), and locally fermented milk product (*n* = 409; 38.3 %) were commonly reported, whereas consumption of raw goat milk was less common (*n* = 184; 17.2 %). Table 2 summarizes the frequency of animal contacts and dietary exposure of the study participants and numbers positive for acute Q fever.

**Table 2:** Frequency of animal contacts and dietary exposure self-reported by febrile patients in Northeastern Kenya

Characteristic	Variable	Total No. patients (%)	Positive for Q fever^a^ (%)	Crude OR (95%CI)	P value
**Contact with goat**	**No**	**378**	**52 (13.70)**	**Ref**	**-**
	**Yes**	**689**	**121 (17.6)**	**4.82 (2.89–8.26)**	**0.003**
Contact with dog	Yes	22	1 (4.5)	Ref	-
	No	1045	172 (16.5)	0.24 (0.03–1.81)	0.267
Contact with sheep	No	675	104 (15.4)	Ref	-
	Yes	392	69 (17.6)	1.17 (0.84–1.64)	0.349
**Contact with cattle**	**No**	**602**	**63 (10.5)**	**Ref**	**-**
	**Yes**	**465**	**110 (23.7)**	**2.65 (1.89–3.72)**	**<0.001**
Contact with camel	Yes	112	13 (11.8)	Ref	-
	No	955	160 (16.7)	0.67 (0.37–1.22)	0.279
**Frequent slaughtering of animals**	**No**	**685**	**68 (9.90)**	**Ref**	**-**
	**Yes**	**382**	**105 (27.5)**	**3.44 (2.46–6.67)**	**<0.001**
**Frequent handling of raw milk**	**No**	**470**	**61 (13.0)**	**Ref**	**-**
	**Yes**	**597**	**112 (18.8)**	**1.55 (1.10–2.18)**	**0.011**
**Contact with birth products** ^**b**^	**No**	**865**	**148 (17.1)**	**Ref**	**-**
	**Yes**	**202**	**25 (12.4)**	**1.43 (0.94–2.19)**	**0.089**
Share house with animals	Yes	98	20 (20.4)	Ref	-
	No	969	153 (15.8)	0.37 (0.08–1.31)	0.239
Frequent contact with wildlife	Yes	2	0 (0)	-	-
	No	1065	173 (16.2)		
Frequently prepare manure	Yes	50	11 (22.0)	Ref	-
	No	1017	162 (15.9)	1.49 (0.44–1.97)	0.258
**Frequently graze animals**	**No**	**350**	**37 (10.6)**	**Ref**	**-**
	**Yes**	**717**	**136 (19.0)**	**1.98 (1.34–2.92)**	**<0.001**
Frequent consumption of raw meat	Yes	57	14 (24.6)	Ref	-
	No	1010	159 (15.7)	0.74 (0.13–3.26)	0.382
**Frequent consumption of raw cattle milk**	**No**	**736**	**57 (7.7)**	**Ref**	**-**
	**Yes**	**331**	**116 (35.0)**	**6.43 (4.52–9.14)**	**<0.001**
**Frequent consumption of raw goat milk**	**No**	**883**	**130 (14.7)**	**Ref**	**-**
	**Yes**	**184**	**43 (23.4)**	**1.77 (1.20–2.61)**	**0.004**
Frequent consumption of raw camel milk	No	124	17 (13.7)	Ref	-
	Yes	943	156 (16.5)	1.24 (0.73–2.14)	0.422
**Frequent consumption of locally fermented milk**	**No**	**658**	**60 (9.1)**	**Ref**	**-**
	**Yes**	**409**	**113 (27.6)**	**3.81 (2.7–5.36)**	**<0.001**
Frequent consumption of animal blood	Yes	45	3 (6.7)	Ref	-
	No	1022	170 (16.6)	0.36 (0.11–1.17)	0.809

*Ref* referent category, *OR* odd ratio, *CI* confidence interval

^a^Patients meeting case definition for acute Q fever by serology or *Coxiella* DNA detection by qPCR

^b^Proportion of patients with recent exposure to aborted materials or help during animal birth

Bold font implies statistically significant results at (*P* < 0.20) that were retained as possible factors and subsequently fitted in the multivariate analysis

### Seroprevalence

A total of 204 (19.1 %, 95 % CI: 16.8–21.6) participants were found to be positive for Q fever based on a parallel interpretation of the three tests applied. Of these, 181 (17.0 %, 95%CI: 14.8–19.4) had anti-*Coxiella* phase II IgG antibodies while 175 (16.4 %, 95%CI: 14.2–18.8) had IgG antibodies against phase I antigens. One hundred ninety-four (18.2 %, 95 % CI: 15.9–20.7 %) patients were positive for IgG antibodies against either phase II or phase I antigens, whilst 162 (15.2 %, 95 % CI: 13.2–17.5) patients were found to be positive for anti-*Coxiella* IgG antibodies against both phase I and phase II antigens (Table 1). Thirteen and eighteen participants tested positive for only IgG phase I and IgG phase II antigens, respectively. One patient remained positive for IgG phase II and borderline for phase I in the repeat tests. Based on the study case definition criteria, acute Q fever was established in a total of 163 (15.3 %, 95 % CI: 13.2–17.6) patients whereas none of the patients had IgG antibody titers against phase I supportive of chronic Q fever infection.

### Molecular detection of Q fever

Quantitative real-time PCR (qPCR) demonstrated *C. burnetii* specific DNA in 2.2 % (10/448) of the examined patients, of which all were serologically negative for Q fever. The tested sera were from patients whose onset of the illness was ≤ 2 weeks and had no antibiotic therapy. The PCR of whole blood or serum is mainly positive within the first two weeks following symptom onset, and becomes negative as the antibody response develops. Detectable seroconversion typically occurs one to three weeks after symptoms appear [39].

Q fever was not clinically suspected by the treating clinicians in any of 173 (16.2 %) positive patients, instead the working diagnosis were mainly presumptive typhoid fever (45.1 %), malaria (6.9 %), pneumonia oracute respiratory infections (37.6 %) and others or fever of unknown origin (FUO) (10.4 %). There was no significant difference in infection found between males and females (*P* = 0.736). Higher seroprevalence (16.7 %, 95 % CI: 14.4–19.3) was found in adults (>18 years) when compared to those aged below (13.0 %, 95 % CI: 8.0–20.2), but the difference was not statistically significant (*P* = 0.283). Somalis were more often seropositive when compared with other ethnic groups (Somali, 17.1 %, 95%CI: 14.8–19.7 and Non-Somali, 7.7 %, 95%CI: 3.6–15.4, *P* = 0.004, Table 1). Among 885 participants who reported regular contact with animals, 160 (18.1 %, 95%CI: 15.7–20.8) tested positive for Q fever whereas only 13 (7.1 %, 95 %: CI: 4.10–12.2) who reported no contact with animals were positive (*P <* 0.001). Most cases of Q fever occurred in full time herders 134 (19.8 %, 95%CI: 16.9–23.1) and livestock trader 15 (25.4 %, 95%CI: 15.4–38.7) when compared to government workers, general merchandise businesses and other occupations (*P <* 0.001). Patients from Wajir county were slightly more seropositive 90 (16.8 %, 95%CI: 13.8–20.3) than those from Garissa county 83 (15.6 %, 95 %: CI: 12.7–19.1) but the difference was not statistically significant (*P* = 0.607). Finally, patients without knowledge of zoonosis 115 (18.6 %, 95%CI: 15.6–21.9, *P =* 0.012) and those without any form of education 130 (19.7 %, 16.8–22.9, *P <* 0.001) were more often seropositive (Table 1).

### Clinical predictors of acute Q fever diagnosis and ‘Q fever’ score

There were 392 (55.4 %) females in the 707 derivation cohort and 188 (52.2 %) females in the 360 validation group. The mean age was 33.6 years (Standard Deviation [SD], 12.2 years) for derivation group and 38.3 years (SD 17.7 years) for the validation group. The mean interval from the onset of fever symptoms until hospital presentation was 14.5 days (SD 10.6 days)/median 8 days for cases in the derivation cohort and 11.9 days (13.2 days)/median 7 days for the validation group. The symptoms and clinical features of the patients are summarized in Table 3. Although the symptoms reported from patients were often combined, Table 3 presents the number of cases in which a particular symptom was reported. Acute Q fever was established in 116 (16.4 %) patients in the derivation group while 57 (15.8 %) patients tested positive in the validation group. Differential diagnosis for selected febrile etiologies was undertaken among the patients, including those classified as with FUO. The tests were selected based on the patients’ clinical syndrome, the most prevalent infections reported to occur in the region and the nature of available laboratory support. These included screening the patients for typhoid fever, malaria, and brucellosis (data not included here). Supportive radiological investigations, hematology and liver enzyme tests were scarcely available and are not presented here.

**Table 3:** Symptoms and clinical features of the febrile patients, Northeastern Kenya

Variable	Total No. (*n* = 707)	Positive for Q fever^a^ (%) (*n* = 116)	Crude OR (95%CI)	P- value
Headache	630	104 (16.5)	1.07 (0.56–2.05)	0.836
Chills	388	64 (16.5)	1.04 (0.68–1.51)	0. 945
Arthralgia/Myalgia	541	87 (16.1)	0.91 (0.57–1.44)	0.673
**Malaise/Fatigue**	**493**	**87 (17.6)**	**1.37 (0.87–2. 15)**	**0.178**
Anorexia	404	62 (15.3)	0.84 (0.56–1.25)	0. 380
**ALRI**	**200**	**61 (30.5)**	**3.61 (2.39–5.44)**	**<0.001**
**Constipation**	**129**	**28 (21.7)**	**1.54 (0.96–2.48)**	**0.074**
Night Sweats	94	12 (12.8)	0.71 (0.38–1.36)	0.308
**Diarrhoea**	**73**	**8 (11.0)**	**0.60 (0.28–1.29)**	**0.081**
Weight loss	62	11 (17.7)	1.11 (0.56–2.20)	0.765
Confusion	15	1 (6.6 %)	0.31 (0.01–7.09)	0.801
Rash	28	6 (21.4)	1.41 (0.56–3.56)	0.466
Vomiting	13	1 (7.7)	0.42 (0.05–3.23)	0.401
**Abdominal pain**	**173**	**37 (21.4)**	**1.57 (1.01–2.42)**	**0.043**
Palpable spleen	91	18 (19.8)	1.31 (0.56–8.69)	0.640
Palpable liver	38	5 (13.1)	0.78 (0.34–1.47)	0.486
**Fever onset (>14 days)**	**253**	**108 (42.7)**	**41.52 (19.78–87.22)**	**<0.001**
Age (>18 years)	618	106 (17.2)	1.64 (0.82–3.26)	0.162
Mean (SD)				
Age (years)	707	33.6 ± 12.2		
Fever onset (days)	707	14.5 ± 10.6		

*ALRI* acute lower respiratory infection, *OR* odds ratio, *CI* 95 % confidence interval, *SD* standard deviation

^a^Patients meeting case definition for acute Q fever by serology or *Coxiella* DNA detection by qPCR

Bolded variables were considered significant (*P* < 0.20) and fitted into multivariate logistic regression model

Univariate analysis of the signs and symptoms revealed that general body malaise and fatigue, ALRI, constipation, abdominal pain, diarrhoea, and fever onset (>2 weeks) were possibly associated with Q fever seropositivity (*P <* 0.20; Table 3).

**Fig. 2:**
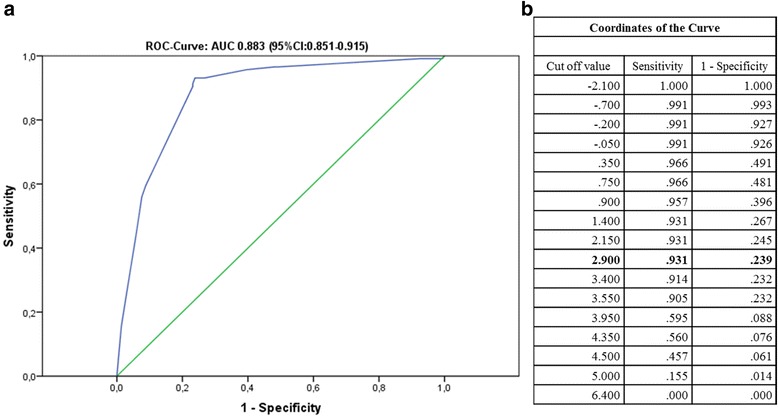
**a** Receiver Operating Characteristics (ROC) and Area Under the Curve (AUC) for assessing the discriminatory capacity of the Q fever score. **b** Cut-off values for the ROC curve

In the final clinical logit model, adjusted for possible confounding variables, ALRI (adjusted Odds ration [aOR]: 2.68, 95%CI: 1.65–4.36), fever onset (>2 weeks) (aOR: 37.59, 95%CI: 17.83–79.27) and abdominal pain (aOR 2.19, 95%CI: 1.02–4.72) were positive predictors of Q fever infection. The analysis revealed that diarrhoea (aOR: 0.34, 95%CI: 0.12–0.96) had predictive value for a negative acute Q fever outcome (*P <* 0.05; Table 4). Using the four variables, predictive score was generated and the scale simplified by assigning values to the nearest one decimal scale (fever onset >2 weeks: +3.6, ALRI: +1.0, abdominal pain: + 0.8 and diarrhoea: −1.1, Table 4). The discriminatory power of the fitted ROC curve area was 0.883 (95%CI: 0.851–0.915, *P <* 0.001, Fig. 2a). An optimal cut-off value of 2.90 (sensitivity 93.1 % and specificity 76.1 %) was selected (Fig. 2b). Only a history of fever (>14 days) singly was a good predictor of positive diagnosis (sensitivity 90.5 % and specificity 76.8 %). The prediction remained strong, if diarrhoea was absent or when at least one of positive predictors was also present. However, none of other clinical predictors (ALRI or abdominal pains) alone or in combination had significant predictive value for positive acute Q fever outcome. Thus, pre-admission fever onset (>14 days) in association with ALRI and abdominal pain and no diarrhoea was the best predictor algorithm for acute Q fever outcome. The prediction rule, reliably identified febrile patients with a score >2.90 as 12.7 times more likely to have a positive acute Q fever test outcome (OR = 12.7; 95 % CI: 4.89–32.72) than the patients with a Q fever score up to 2.90.

**Table 4:** Result of multivariate analysis showing significant clinical predictors for acute Q fever and calculation of Q fever score

Variable	Crude OR (95%CI)	aOR (95%CI)	β Coefficient	P- value
ALRI				
No	Ref	Ref		-
Yes	3.61 (2.39–5.44)	2.68 (1.65-4.36)	0.986	<0.001
Abdominal pain				
No	Ref	Ref		-
Yes	1.57 (1.01–2.42)	2.19 (1.02–4.72)	0.788	0.004
Diarrhoea				
No	Ref	Ref		-
Yes	0.60 (0.28–1.29)	0.34 (0.12–0.96)	−1.075	0.042
Fever onset (>14 days)				
No	Ref	Ref		-
Yes	41.52 (19.78–87.22)	37.59 (17.83–79.27)	3.627	<0.001
H-L test				0.567
ROC (AUC)	0.883 (0.851–0.915)			<0.001
Q fever score	Q fever score (rounded) = 1.0x(ALRI) + 3.6x(Symptoms onset (>14 days) + 0.8x (Abdominal pains) -1.1x(Diarrhoea).			

*ALRI* acute lower respiratory infection, *Ref* referent category, *aOR* adjusted odds ratio, *CI* 95 % confidence interval, *H-L test* Hosmer-Lemeshow goodness- of-fit test, *ROC* receiver operating characteristics, *AUC* area under the curve

### Risk factor analysis for acute Q fever

Univariate analyses of demographic, social-economic and dietary characteristics showed no significant associations with seropositivity between gender, county of residence of the patient or across different age strata, and occupations except herders (OR; 2.96, 95 % CI: 1.15–8.34) and livestock traders (OR; 4.09, 95 % CI: 1.26–13.27) who had significantly higher odds for seropositivity. Having secondary education or above (OR; 0.39, 95 % CI: 0.17–0.81) and knowledge about zoonosis (OR; 0.78 CI: 0.36–0.99) were associated with decreased risk of Q fever seropositivity. Odds of Q fever seropositivity were significantly higher for patients of Somali ethnicity (OR; 1.92, 95 % CI: 1.29–3.76).

Regular contact with cattle (OR; 2.65, 95 % CI: 1.89–3.72) or goats (OR; 4.82, 95 % CI: 2.89–8.26) were independently associated with Q fever seropositivity. The risk for *Coxiella* infection was higher for those who reported the following: contact with aborted materials or assist in animal births (OR; 1.43, 95 % CI: 0.94–2.19), handling raw milk (OR; 1.55, 95 % CI: 1.10–2.18), grazing animals (OR; 1.98, 95 % CI: 1.34–2.92), and slaughtering of animals (OR; 3.44, 95 % CI: 2.46–6.67). Q fever seropositivity was significantly associated with consumption of raw cattle milk (OR; 6.43, 95 % CI: 4.52–9.14), raw goat milk (OR; 1.77, 95 % CI: 1.20–2.61) and locally fermented milk products (susac) (OR; 3.81, 95 % CI: 2.73–5.36) (Table 2).

In the final combined multivariate analyses, significant association persisted for pre-admission fever (>14 days) (aOR; 36.35, 95 % CI: 19.84–69.49) and ALRI (aOR; 2.41, 95 % CI: 1.53–6.36), while abdominal pain (aOR; 1.95, 95 % CI: 1.28–3.58) association with seroposivity was strengthened.

Regular contact with cattle (aOR: 2.09, 95 % CI: 1.73–5.98), or goats (aOR: 3.74, 95 % CI: 2.52–9.40) and frequent slaughter of animals (aOR: 1.78, 95 % CI: 1.09–2.91) remained with significantly higher odds of seropositivity. The dietary exposures significantly associated with seropositivity in the final combined model included: consumption of raw cattle milk (aOR: 2.49, 95%CI: 1.48–4.21) and locally fermented milk products (aOR: 1.66, 95%CI: 1.19–4.37).

The odds of Q fever were lower for patients who presented with diarrhoea (aOR; 0.63, 95 % CI: 0.38–1.00) and for those with secondary education or above at the time of hospital visit (aOR; 0.71, 95 % CI: 0.24–0.96) (Table 5).

**Table 5:** Results of final combined logistic regression analysis showing risk factors found associated with Q fever seropositivity in febrile patients, northeastern Kenya

Risk factor	Variable	Crude OR	P- value	aOR(95%CI)	P- value
Age Groups (years)	<19	Ref	-	Ref	-
	>19–29	0.70 (0.37–1.35)	0.282	1.16 (0.63–3.92)	0.178
	30–39	1.52 (0.86–2.65)	0.145	1.86 (0.55–8.26)	0.433
	40–49	1.73 (0.97–3.10)	0.063	2.03 (0.87–4.38)	0.609
	>50	1.28 (0.66–2.48)	0.458	1.21 (0.51–2.97)	0.124
Gender	Female	Ref	-	-	-
	Male	0.95 (0.68–1.31)	0.736		
Residence county	Garissa	Ref	-	-	
	Wajir	1.09 (0.78–1.51)	0.607		
Higher education	Yes	Ref	-	Ref	-
	No	0.39 (0.17–0.81)	<0.001	0.71 (0.24–0.96)	0.038
Ethnicity	Other	Ref	-	-	-
	Somali	1.92 (1.29–3.76)	0.004		
Zoonosis knowledge	No	Ref		-	-
	Yes	0.78 (0.36–0.99)	0.025		
Occupation	Others	Ref	-	-	-
	Livestock trader	4.09 (1.26–13.27)	0.019		
	Student	1.05 (0.26–4.14)	0.941		
	General merchandise	1.06 (0.28–3.96)	0.932		
	Civil servant	0.81 (0.39–2.76)	0.739		
	Herder	2.96 (1.15–8.34)	0.041		
Animal Contact	Cattle				
	No	Ref	-	Ref	-
	Yes	2.65 (1.89–3.72)	<0.001	2.09 (1.73–5.98)	0.004
	Goats				
	No	Ref	-	Ref	-
	Yes	4.82 (2.89–8.26)	0.003	3.74 (2.52–9.40)	0.006
	Frequent slaughter of animals				
	No	Ref	-	Ref	-
	Yes	3.44 (2.46–6.67)	<0.001	1.78 (1.09–2.91)	0.021
	Contact with birth products^a^	Ref	-	-	-
		1.43 (0.94–2.19)	0.089		
	Frequent handling of raw milk	Ref	-	-	-
		1.55 (1.10–2.18)	0.011		
	Frequently graze animals	Ref	-	-	-
		1.98 (1.34–2.92)	0.030		
Dietary contact	Frequent consumption of raw cattle milk				
	No	Ref	-	Ref	-
	Yes	6.43 (4.52–9.14)	<0.001	2.49 (1.48–4.21)	0.001
	Frequent consumption of locally fermented products				
	No	Ref	-	Ref	-
	Yes	3.81 (2.73–5.36)	<0.001	1.66 (1.19–4.37)	0.014
	Frequent consumption of raw goat milk				
	No	Ref	-	-	-
	Yes	1.77 (1.20–2.61)	0.004		
	ALRI				
	No	Ref	-	Ref	-
	Yes	3.61 (2.39–5.44)	<0.001	2.41 (1.53–6.36)	<0.001
Clinical	Abdominal pain				
	No	Ref	-	Ref	-
	Yes	1.57 (1.01–2.42)	0.004	1.95 (1.08–3.58)	0.019
	Diarrhoea				
	No	Ref	-	Ref	-
	Yes	0.60 (0.28–1.29)	0.042	0.63 (0.38–1.00)	0.047
	Fever onset (>14 days)				
	No	Ref	- < 0.001	Ref	-
	Yes	41.52 (19.78–87.22)		36.35 (19.84–69.49)	<0.001
H-L test					0.412
ROC (AUC)				0.718 (0.673–0.784)	<0.001

*Ref* referent category, *aOR* adjusted odds ratio, *CI* confidence interval, *H-L test* Hosmer-Lemeshow goodness- of-fit test, *ROC* receiver operating characteristics, *AUC* area under the curve

^a^Proportion of patients with recent exposure to aborted materials or helped during animal birth

## Discussion

This cross-sectional study from a pastoralist community in Kenya found an unexpectedly high prevalence (16.2 %) of acute Q fever in febrile patients who were not suspected of Q fever infection during hospital diagnosis. Instead these patients were mainly suspected for the common tropical fevers or fever of unknown origin. Previous studies in Kenya have found that a considerable proportion of febrile patients still continue to be treated for presumptive malaria and blood stream infections using antimalarials and penicillin based antibiotics, respectively [40–42]. However, these treatments have no activity against *C. burnetii.* Though not directly assessed in the present study, our findings strongly suggest that patients with Q fever were likely to leave hospital without the specific treatment for Q fever. These results taken together with previous studies elsewhere in Africa [27, 40, 41, 43, 44], provide further evidence that in absence of clear guidelines for the management of febrile illness and incorporation of reliable diagnostic tests in the hospitals to enable accurate fever diagnosis, clinicians continues to encounter missed opportunities to accurately detect and treat other causes of fever. The patients thus don’t benefit from appropriate antibiotic therapy.

The prevalence of Q fever in this study compare to that reported in similar studies in Burkina Faso (13.1 %) [45] and Egypt (12 %) [46], but the seroprevalence was considerably higher than that reported in Tunisia (8 %) [27], Mali (5 %) [26] and Tanzania (5 %) [43]. Similarly, our results do not correspond to the findings of studies conducted in Croatia (27.5 %) [47], Iran (35.2 %) [48] and Turkey (36 %) [49], respectively. However, it is difficult to compare findings of studies done in different countries because of the different sampling criteria, selected study population, and the different laboratory tests and cut-offs used that may affect the outcomes.

We found no significant difference in infection among individual age groups or sex. This is dissimilar to previous studies in different (high-income) settings [1, 50, 36] which suggest that older persons (25–60 years) and men are at a greater risk of infection due to cumulative risk of exposure and men dominance in risky occupations. In contrast, a study by Muga *et al*. [15] described nomadic pastoralism in Kenya as highly labor-intensive and work is shared out almost entirely by all household members (including children above four years). This implies that the entire population may be at risk of exposure to infected animals or materials very early in life.

In our study, a new prediction score (Q fever score) reliably differentiated acute Q fever infection in febrile patients with undifferentiated illness. With a cut off value of 2.90 (sensitivity 93.1 % and specificity 76.1 %), the best diagnostic algorithm; history of fever (>14 days) singly without diarrhoea, or in associated with ALRI and/or abdominal pains had the best predictive value for positive acute Q fever outcome. On the contrary, ALRI or abdominal pains present alone or in combination did not meet the threshold predictive of infection and especially when associated with diarrhoea. To our knowledge, this is the first study to evaluate such prediction rule for the diagnosis of acute Q fever. Whereas, previous studies designed to assess clinical and laboratory features of Q fever patients reported hepatitis and pneumonia as common clinical presentations, the authors did not propose a clinical prediction rule that could be of value during initial patient diagnosis [51, 52]. Other studies have assessed the sensitivity and specificity of individual signs and symptoms to establish clinical diagnosis of febrile etiologies such as typhoid fever [53], malaria [54, 55] and brucellosis relapse [56] in developing countries. Similar to our study, none of the constitutional symptoms proved a good predictor of infection. In our study, the new Q fever score demonstrated that patients with a score above the cut-off value of 2.90, were 12 times more likely to test positive for acute Q fever than those with a score upto the cutoff (OR = 12.7; 95 % CI: 4.89-32.72, *P < 0.001*).

Within the past decade, malaria has substantially declined in many endemic countries [57]. This together with the increasing availability and use of malaria rapid diagnostic tests to exclude malaria have led substantial adherence to the WHO recommended “test and treat” policy. However, a growing body of evidence in Africa shows that clinicians are presently faced with a growing proportion of patients with severe febrile illness from previously under-recognized zoonotic diseases but tools to guide subsequent detection and management are often lacking [16, 17, 24–27, 40, 43]. Under these circumstances, the present new Q fever score would be of particular value to practicing clinicians working in resource limited countries in making decisions on patients necessitating meticulous attention in regard to Q fever during initial diagnosis and empiric treatment. For instance, using the score supported by screening patients for the risky epidemiological factors would be useful to clinicians to make clinical decisions on when to prefer prescribing doxycycline versus the commonly used penicillin based drugs during empiric treatment of fever of unknown origin in such underdeveloped areas. However, we acknowledge that this score may not provide a definitive clinical algorithm for acute Q fever detection because of the limitations of the present study, but a clinically supportive guide for clinicians.

We report for the first time the correlates of socio-economic and dietary practices with Q fever seropositivity in Kenya. We found higher odds of seropositivity in individuals who reported regular occupational or domestic contact with goats, cattle and those involved in regular slaughter of animals, thus highlighting the significant role of livestock contact in transmission of *C. burnetii*. Slaughtering of animals (usually uninspected by health officials) is a common cultural practice of communities in the study area e.g. during Eid-al-Adha, dowry payment, and wedding ceremonies or during the regular diet preparation [15, 58]. During these events, individuals may be exposed to a high risk of contracting Q fever. Our results correspond well with previous studies in different countries that found higher seroprevalences in persons who were in close contact with cattle and goats. In addition, consumption of raw cattle milk (aOR: 2.49) and the locally fermented milk products i.e. susac (aOR: 1.66) were important risk factors. This practice is also common among the pastoralist communities in the study area because of strong rooted cultural norms that associate unboiled milk with high nutrition value [31]. Indeed, most of the patients of Somali ethnic group were unaware that drinking raw camel or cattle milk was capable of causing diseases and thought that boiling was not necessary. These practices may present significant risk of exposure to milk borne pathogens as milk from different animals is pooled before consumption or fermented into the traditional products (susac) without a pre-heating step. Previous epidemiological studies have also described strong associations between consumption of raw milk and *C. burnetii* seropositivity [52, 59], but the risk of oral transmission is still widely considered as minimal compared to aerosol route [60, 61]. Therefore, this evidence can be strengthened by more research to definitively demonstrate the probability of infection through oral route and determination of pathogens dose capable of causing the disease.

### Limitations

A potential limitation in our findings is that our study was hospital based where the population under investigation was febrile patients seeking treatment. Therefore, the afebrile cases or those that were unable to present to the hospitals were inevitably not captured by our sampling strategy. Again, the patient samples were generated in absence of a robust probabilistic sampling method. This has the effect of limiting the generalization of the results. A population-based survey or follow up of patients to obtain a convalescent sera sample was not feasible due to the nomadic lifestyle of the patients, the ongoing inter-clans conflicts and militia activities in the region. However previous studies have reported adequate performance of the used commercial serion ELISA classic test kits in detection of phase II specific IgG and IgM [36, 62] respectively, in diagnosis of acute Q fever. The novelty of the present study was that the ELISA positive sera were further confirmed with the reference diagnostic method (IFA) and the patients whose fever onset was ≤ 2 weeks were further screened for Q fever using a quantitative real-time PCR. Nevertheless, the possibility of recent infection cases, false positive or chronic cases being considered as acute Q fever cannot be completely rule out.

The utility of our prediction score should be interpreted in the context of potential limitation. The cross-sectional nature of the study did not allow more comprehensive evaluation and follow up of each patient and the fact that the prediction rule was derived by logistic regression modeling which can be prone to residual confounding effects. Another potential limitation is related to the heterogeneous clinical presentation of Q fever in patients and the fact that the score was validated in patients from the same region where it was developed. Though deliberate efforts were made to exclude the commonly reported febrile etiologies in the region including typhoid fever, malaria and brucellosis, unfortunately due to the nature of the present study and scarcity of appropriate laboratory and radiological support, a comprehensive differential diagnosis for other febrile etiologies among our patients was not feasible. These factors may have affected the discriminatory index of the present score. Nevertheless, our study was not designed to give a definite clinical algorithm but one to help clinicians in distinguishing febrile cases probably due to Q fever from febrile episodes not due to Q fever and aid in making clinical empiric therapeutic decisions. Therefore, validation of the present prediction score in well-designed studies in diverse independent cohorts is highly warranted.

### General recommendations

A one health approach for surveillance of emerging and remerging infectious diseases should be encouraged in Kenya to support design and implementation of rational control strategies for febrile diseases. Education programmes and appropriate preventive interventions targeting the significant risk factors needs to be designed for the communities at high risk of exposure. Increased clinician and laboratory personnel awareness and access to laboratory testing capacity is needed to enable timely detection and early treatment to prevent severe sequelae.

## Conclusions

This is the first epidemiological study to report Q fever as a serious public health problem in Northeastern Kenya and a description of social-cultural, occupational, and dietary factors influencing human exposure. We present a simple predictive score based on clinical features. A Q fever score supported by screening patients for the risky epidemiological factors will ultimately help clinicians in making clinical judgments in distinguishing febrile illness probably due to Q fever from other etiologies and selection of appropriate therapeutic decisions (e.g. penicillin based antibiotics versus doxycycline for empiric treatment), particularly when no microbiological testing is available. The findings also provide a framework to initiate well-designed research in linked animal and human populations in different ethnic entities, agricultural production and management systems in Kenya.

## Abbreviations

AFI, acute febrile illness; ALRI, acute lower respiratory infections; ASAL, arid and semi-arid land; CDC, Centers for Disease Control and Prevention; EFSA, European Food Safety Authority; ELISA, enzyme-linked immuno sorbent assay; FUO, fever of unknown origin; IFA, indirect immunofluorescence assay; IHC, immunohistochemistry; PCR, polymerase chain reaction; ROC, receiver operating characteristics; WHO, World Health Organization.
